# A case of multiorgan IgG4-related disease with coronary artery vasculitis: Review of the 2019 Rheumatologic Classification Guidelines and what radiologists need to know

**DOI:** 10.1016/j.radcr.2025.07.022

**Published:** 2025-08-05

**Authors:** Jasmine Zhao, Asmar Ghani, Ali Mohammadzadeh, Cato Chan, James Shi

**Affiliations:** Department of Radiology, University of California Irvine, University of California Irvine Medical Center, Orange, CA, USA

**Keywords:** IgG4-related disease, Coronary arteritis, Sclerosing cholangitis, Renal disease, Retroperitoneal fibrosis, Autoimmune pancreatitis

## Abstract

Immunoglobulin G4-related disease (IgG4-RD) is a rare fibroinflammatory disorder with diverse manifestations that can affect nearly any organ system. This case report describes a 58-year-old man presenting with obstructive jaundice due to a biliary stricture concerning for cholangiocarcinoma; however, eventual pathology, serology and imaging findings confirmed IgG4-RD. Over 2 years, the disease progressed to involve multiple organs, including 3-vessel coronary arteritis, a serious but underrecognized manifestation that may present with the ``pigs-in-a-blanket” sign on imaging. Despite treatment, the patient ultimately suffered a fatal cardiac event soon after. This case underscores the importance of recognizing characteristic imaging features, particularly those outlined in the 2019 American College of Rheumatology/European League Against Rheumatism classification criteria, which can act as a framework for radiologists and clinicians in early diagnosis. Prompt recognition, multidisciplinary collaboration, and timely treatment are essential to avoid unnecessary surgical interventions and reduce the risk of life-threatening complications.

## Graphical abstract

**Figure:**
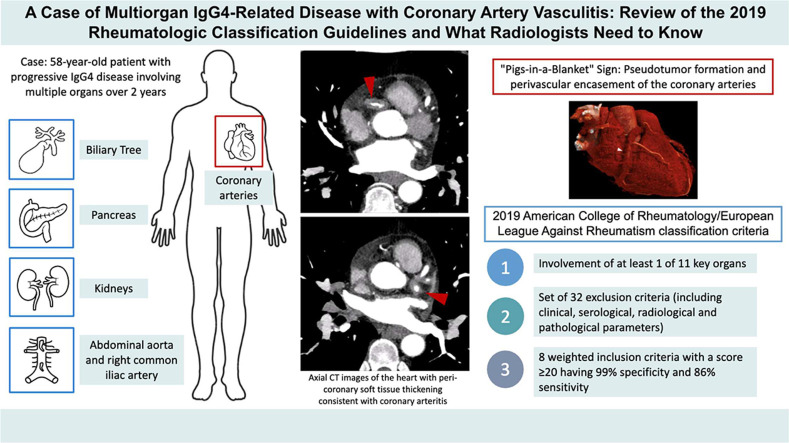

## Introduction

Immunoglobulin G4-related disease (IgG4-RD) is a rare fibroinflammatory disorder that can affect virtually any organ. It is characterized histologically by IgG4-positive lymphoplasmacytic infiltrates, storiform fibrosis, tumefactive lesions, obliterative phlebitis, and elevated serum IgG4 levels [1,2]. First recognized as a distinct clinical entity in 2003, IgG4-RD has since been identified across numerous organ systems, with a predilection for the pancreas, bile ducts, salivary glands, orbits, kidneys, lungs, aorta, retroperitoneum, meninges, and thyroid [[2], [3], [4]].

Although the infrarenal aorta is often involved in IgG4-RD, coronary artery vasculitis remains a rare and potentially under-recognized manifestation [5]. When present, it may appear as diffuse wall thickening, luminal narrowing and has been associated with serious complications such as myocardial infarction, arrhythmia, and sudden cardiac death. A recent case series has emphasized the higher prevalence in men and vasculopathy as the most common cardiovascular complication [6].

Currently, the pathogenesis of IgG4-RD remains incompletely understood with evidence suggesting a dysregulated immune response involving T-helper 2 and regulatory T cells, driving IgG4-producing plasma cells [4]. The disease primarily affects middle-aged to older men, with an estimated male-to-female ratio of 2:1 [1,2]. Clinical presentations often mimic malignancies or autoimmune conditions such as Sjögren’s syndrome, vasculitis, or systemic lupus erythematosus, complicating diagnosis.

Early recognition is essential to avoid unnecessary surgical interventions and to initiate timely immunosuppressive therapy. To support epidemiological studies and standardize research cohorts, the 2019 American College of Rheumatology/European League Against Rheumatism (ACR/EULAR) proposed a classification system for IgG4-RD derived from a cohort of almost 1900 patients. These criteria incorporate organ-specific involvement, serologic, histopathologic, and imaging features, along with exclusion criteria to differentiate IgG4-RD from mimickers [3]. In validated cohorts, a score of 20 or more on the ACR/EULAR classification system demonstrates high diagnostic accuracy for IgG4-RD with approximately 99% specificity [3]. Failure to meet the classification criteria should not preclude appropriate diagnosis and management in the appropriate clinical context.

In this case, we report a unique case of progressive multiorgan IgG4-RD culminating in severe coronary artery vasculitis and fatal cardiopulmonary arrest. This case highlights the need for prompt recognition and multidisciplinary management to mitigate the risk of irreversible or fatal complications.

## Case report

A 58-year-old man with no significant past medical history initially presented to an outside hospital with 3 weeks of progressively worsening right upper quadrant abdominal pain, decreased oral intake, nausea, vomiting, and dark urine. Physical examination revealed scleral icterus. Laboratory evaluation showed a total bilirubin of 15.9 mg/dL (reference range: 0.2-1.2 mg/dL), alkaline phosphatase (ALP) of 516 U/L (reference range: 34-104 U/L), aspartate aminotransferase (AST) of 168 U/L (reference range: 10-25 U/L), and alanine aminotransferase (ALT) of 166 U/L (reference range: 9-46 U/L).The computed tomography (CT) scan demonstrated mild intrahepatic and extrahepatic biliary ductal dilatation with abrupt narrowing at the pancreatic head.

Endoscopic ultrasound and endoscopic retrograde cholangiopancreatography (EUS/ERCP) at the outside facility revealed a long-segment 1.2 cm distal common bile duct (CBD) stricture with abrupt termination near the pancreatic head. A biliary sphincterotomy was performed and a stent was placed. Brush cytology was negative for malignancy, though cholangiocarcinoma remained a concern given the limited sensitivity of cytology and an elevated CA 19-9 level of 109 U/mL (reference range: <34 U/mL). Following the procedure, liver enzymes improved, and the patient was discharged with antibiotics and plans for a Whipple procedure due to ongoing concern for malignancy.

**Fig. 1:**
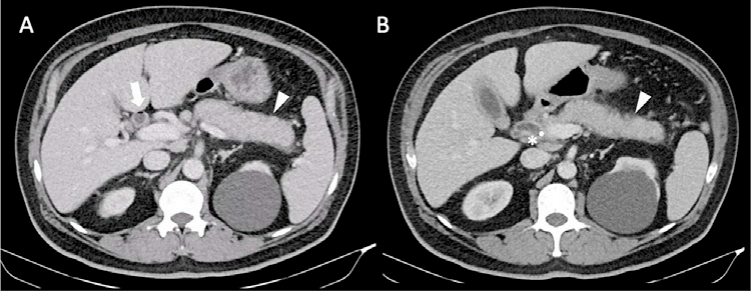
Axial CT images of the abdomen with diffuse enlargement of the pancreas, loss of clefts, and minimal peri-pancreatic stranding (white arrowhead). There is also thickening of the common bile duct wall with trace pneumobilia (white arrow) in setting of biliary stent placement (asterisk). Mild thickening of the gallbladder wall is also seen in (B) without cholelithiasis.

Two months later, the patient came to our emergency department with abdominal pain, fever, and decreased urine output. At that time, bilirubin, transaminases, and lipase had normalized, but ALP remained elevated at 270 U/L. CT imaging showed persistent mild biliary dilation, circumferential CBD wall thickening, and pancreatic enlargement with heterogeneous enhancement and subtle peripancreatic stranding. These findings suggested acute interstitial pancreatitis, although autoimmune pancreatitis (AIP) and IgG4-related pancreatitis were mentioned as differential diagnoses (Fig. 1). He was discharged with close outpatient follow-up.

Due to persistent concern for malignancy, the patient elected to proceed with surgery. One month later, he underwent a Whipple procedure with partial gastrectomy, small bowel resection, Roux-en-Y enteroenterostomy, choledochojejunostomy, and pancreatic biopsy at the outside hospital. Intraoperatively, the CBD appeared thickened and inflamed. Pathology showed no evidence of malignancy, and positive IgG4 staining was reportedly identified in the pancreatic biopsy, although the final pathology report was not available to our institution, leading to diagnostic delay.

Following surgery, the patient was followed at our institution’s outpatient clinic for almost a year, reporting intermittent abdominal pain, fever, and myalgias. Laboratory workup revealed a markedly elevated serum IgG4 level of 1413 mg/dL (reference range: 4-86 mg/dL), positive anti-smooth muscle antibody (1:80) (reference range: <1:20), CA 19-9 of 49 U/mL, and a low fecal elastase level of 33 µg/g (reference range: >200 µg/g). He was referred to gastroenterology, but the etiology of the biliary stricture remained unclear. Given difficulty in obtaining the outside pathology report, both choledocholithiasis and IgG4-RD were considered possible causes of stricture formation. Hepatobiliary surgery consultants expressed greater suspicion for IgG4-RD and recommended expedited rheumatology evaluation and repeat ampullary biopsies with EUS.

A repeat EUS demonstrated diffusely hypoechoic pancreatic parenchyma. However, IgG4 immunostaining of the ampullary samples obtained during the procedure was noncontributory due to extensive nonspecific background staining. At this time, the diagnosis of IgG4-RD was equivocal, as histopathologic confirmation was still pending, and pancreatic involvement remained questionable. When assessed using the ACR/EULAR criteria, the patient met a total of 11 points given serum IgG4 levels were greater than 5 times the upper limits of normal.

**Fig. 2:**
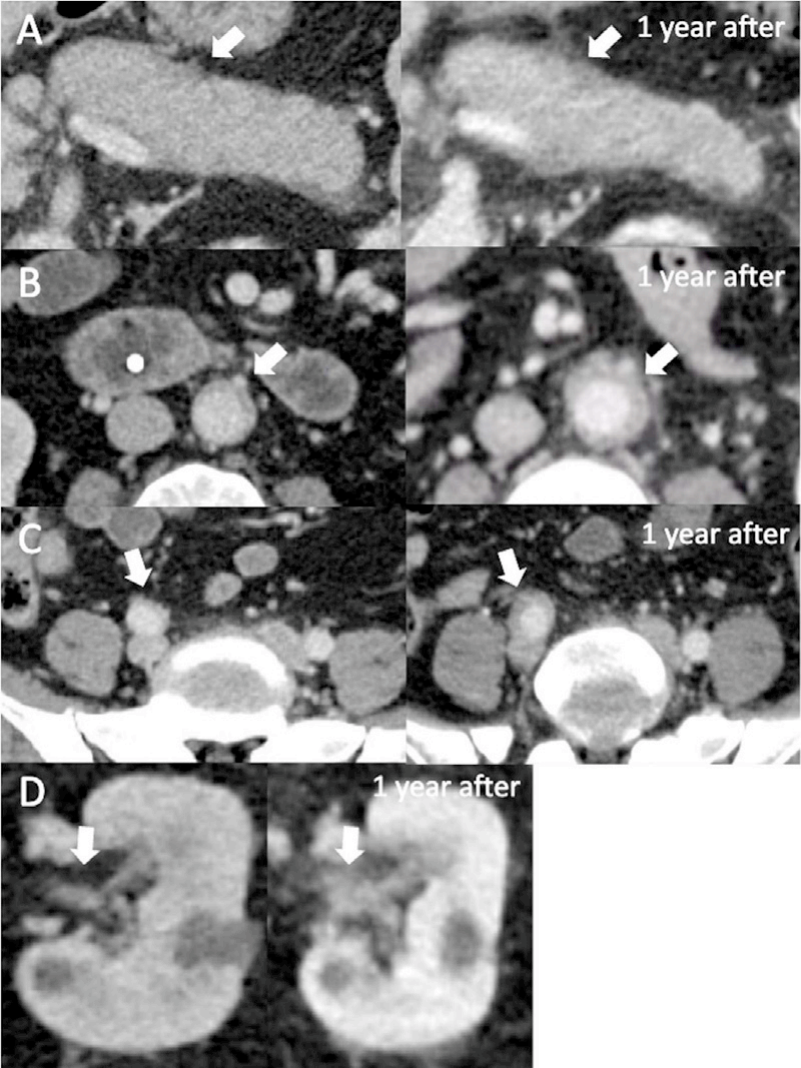
(A) Axial CT imaging dated 1 year apart with increased ill-defined halo of soft tissue attenuation surrounding the pancreas (white arrow). (B) Axial CT imaging dated 1 year apart with increased soft tissue thickening around the abdominal aorta (white arrow). (C) Axial CT imaging at the level of the pelvis with soft tissue thickening around the right iliac artery (white arrow). (D) Axial CT imaging that is dated 1 year apart with increased thickening of the left renal pelvis (white arrow).

The rheumatologist ordered repeat CT imaging, and another attempt was made to obtain the outside pathology slides of the pancreatic biopsy from the Whipple procedure. Slides obtained a month later showed positive IgG4 immunostaining. Furthermore, the repeat CT findings were consistent with progressive, multiorgan involvement of IgG4-RD. Chest CT identified multivessel coronary artery perivascular soft tissue thickening and luminal narrowing. CT of the abdomen and pelvis identified pancreatic enlargement with capsule-like rim, bilateral renal pelvic thickening, and progression of arteritis involving the infrarenal aorta and right common iliac artery (Fig. 2). Even without the positive immunostaining and serum IgG4 levels, the radiologic findings alone totaled up to 27 points using the ACR/EULAR criteria, confirming the diagnosis of IgG4-RD.

**Fig. 3:**
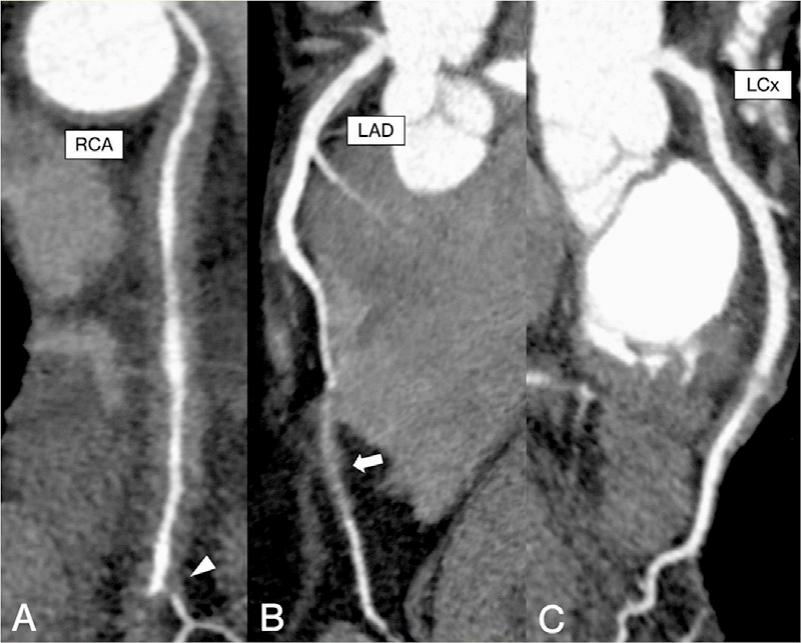
Curved MPR reconstructions of the (A) right coronary artery (RCA) to posterior descending artery (PDA), (B) left anterior descending (LAD), (C) and left circumflex (LCx) demonstrate multifocal coronary pseudotumor formation causing severe stenosis of the PDA (white arrowhead) and distal LAD (white arrow).

**Fig. 4:**
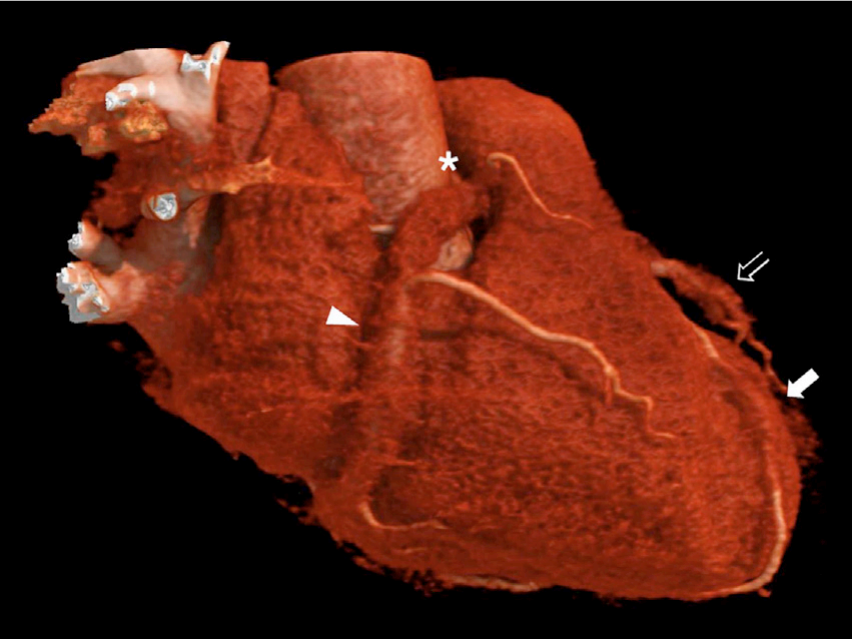
3D CT reconstruction demonstrates coronary pseudotumor formation and encasement (“pigs-in-a-blanket” sign) of the right coronary artery (RCA) (white arrowhead), third diagonal branch (outline of white arrow), and distal left anterior descending artery (white arrow). Note the anomalous interatrial course of the RCA between the aorta and the pulmonary artery (asterisk).

Shortly after the CT findings, the patient was referred to the emergency department and admitted due to concerns for active coronary artery vasculitis. The patient reported being generally asymptomatic but had infrequent chest pain over the past few months. He was initiated on high-dose intravenous methylprednisolone (48 mg/day) and rituximab (1000 mg). Echocardiography demonstrated a preserved ejection fraction (57%) with no regional wall motion abnormalities. A dedicated coronary CT angiogram showed triple-vessel concentric perivascular soft tissue thickening with moderate stenosis of the right coronary artery and severe stenosis of the posterior descending artery and diagonal branches (Fig. 3, Fig. 4). The anomalous origin of the right coronary artery (ARCA) from the left coronary cusp with slit-like orifice and acute angle takeoff was incidentally noted.

The cardiologist recommended deferring immediate revascularization, emphasizing the need to reduce the underlying inflammatory process. A repeat coronary CTA was planned to reassess the severity of stenosis following steroid treatment. Planned outpatient evaluation included a cardiac stress test and referral to a congenital heart specialist for assessment of the ARCA. The patient was discharged after 2 days on a tapering course of oral corticosteroids. Unfortunately, 3 days after discharge, he experienced sudden-onset chest pain and suffered a cardiopulmonary arrest at home. Despite resuscitation efforts, he was pronounced dead on arrival at the hospital.

## Discussion

IgG4-RD is an immune-mediated, multi-organ condition with a wide spectrum of clinical manifestations. Its diverse presentations often pose diagnostic challenges due to overlap with malignancies (e.g., cholangiocarcinoma, pancreatic cancer, lymphoma), infections, vasculitides, and other immune-mediated disorders [1,2,7]. Although elevated serum IgG4 levels can support the diagnosis, up to 20-30% of patients may have normal levels [3,8]. Additionally, certain sites of involvement like the retroperitoneum are less frequently associated with elevated serum IgG4 levels [8,9]. While AIP is classically linked to IgG4-RD, it can also occur as an isolated entity. This case underscores how the variable manifestations of IgG4-RD can delay diagnosis and, in some cases, lead to unnecessary or invasive interventions.

The 2019 ACR/EULAR classification system provides a structured framework for evaluating a potential diagnosis of IgG4-RD, utilizing a 3-step approach. First, the patient must exhibit involvement of at least 1 of 11 organs in a pattern consistent with IgG4-RD. Second, 32 exclusion criteria, spanning clinical, serologic, radiologic, and pathologic findings, must be assessed; the presence of any exclusion criterion rules out the diagnosis. Third, 8 weighted inclusion domains are scored across clinical, serological, radiological, and pathological features. A total score of ≥20 points has shown approximately 99% specificity and 86% sensitivity for classifying IgG4-RD in a validated cohort, although the threshold for biopsy remains low for accessible sites [3]. Table 1, Table 2 outline the imaging-specific inclusion and exclusion criteria.

**Table 1:** Radiological exclusion guidelines adapted from the 2019 American College of Rheumatology/European League Against Rheumatism classification system for IgG4-related disease.

Radiological exclusion criteria
Rapid progression on imaging (within 4 to 6 weeks).
Long bone abnormalities consistent with Erdheim-Chester Disease.
Splenomegaly (over 14 cm without alternative etiology).
Suspicious lesions for malignancy or infection that have not been adequately investigated.

**Table 2:** Radiological inclusion guidelines adapted from the 2019 American College of Rheumatology/European League Against Rheumatism classification system for IgG4-related disease.

Radiological inclusion criteria		
Organ system	Findings	Points
Head and neck		
*Lacrimal or salivary gland (typically bilateral, but can be unilateral)*	• One set of glands • Two or more set of glands	+6 +14
Chest		
	• Peribronchovascular and septal thickening • Paravertebral band-like soft tissue in the thorax	+4 +10
Abdomen and pelvis		
*Pancreas and biliary tree*	• Diffuse pancreatic enlargement (>2/3 of pancreas) • Capsule-like rim with decreased enhancement • Biliary tree involvement (smooth wall thickening, sclerosing cholangitis)	+8 +11 +19
		
*Kidneys*	• Renal pelvis thickening • Low-density patchy or round renal cortical lesions	+8 +10
		
*Retroperitoneum*	• Diffuse aortic wall thickening • Circumferential/anterolateral soft tissue around infrarenal aorta or iliac arteries	+4 +8

Although the ACR/EULAR criteria were initially created for clinical research purposes, it has been validated in large patient cohorts and there is merit in using it as a structured framework to guide clinical evaluation and reasoning. The criteria emphasize the importance of integrating diagnostic data, and systematically excluding mimicking conditions such as malignancy, vasculitis, and infection. This approach aligns with best clinical practices and can help ensure a thorough and reproducible process, especially in complex or multisystem cases [10,11]. In a published case series by Ratwatte et al., for instance, the ACR/EULAR criteria were applied and used to diagnose a patient who initially presented with pericarditis and found to have elevated serum IgG4 (between 2 and 5x normal), diffuse pancreatic tail enlargement, aortic wall thickening, and perinephric cortical thickening. Although no biopsy was performed, the diagnosis of IgG4-RD was made according to the criteria and the patient experienced symptomatic improvement after steroids [12].

While large vessels like the aorta are more commonly affected in IgG4-RD, coronary artery vasculitis is exceptionally rare and not included in the ACR/EULAR criteria. In a cohort of 361 patients, only 4% had coronary artery involvement, which included aneurysmal dilation, arterial wall thickening, periarterial soft tissue encasement, stenosis, and calcification [5]. Among the imaging features, the “pigs-in-a-blanket” sign is particularly characteristic, referring to circumferential, mass-like soft tissue encasing the coronary arteries, resembling a sausage wrapped in dough [[13], [14]]. Analogously, the “mistletoe sign” refers to enhancing soft tissue masses surrounding the coronary arteries, resembling clusters of mistletoe leaves on tree branches. This finding is associated with coronary artery involvement in idiopathic retroperitoneal fibrosis (Ormond’s disease) [15]. However, there are several case reports of IgG4-related retroperitoneal fibrosis, resulting in a ``mistletoe” appearance of the coronary arteries [16]. It may be difficult to distinguish from the “pigs-in-a-blanket” sign, as perivascular pseudotumor formation with arterial encasement can result from a range of nonspecific inflammatory processes. However, such encasement can lead to critical vascular stenosis, predisposing to myocardial ischemia, infarction, and, in rare cases, sudden cardiac death [17].

The presence of IgG4-related coronary artery vasculitis becomes more complicated when coexisting with congenital anomalies. In this case, the ARCA with interarterial course between the aorta and pulmonary is a variant that occurs in approximately 0.23% of the general population [18]. To our knowledge, no prior reports describe the coexistence of both ARCA and IgG4-coronary artery vasculitis, reflecting the rarity of each condition. The hemodynamic implications of this congenital anomaly in the setting of IgG4-RD are difficult to determine, but a compounding effect cannot be excluded. The risk of cardiac death is highest in young individuals with interatrial anomalous left coronary artery (ALCA) during strenuous exertion. Studies of adult cohorts with ARCA undergoing conservative therapy have very low mortality (<1%) after 1.3 to 5.6 years of follow-up [19,20]. According to the American Heart Association and American College of Cardiology, the management of patients with interarterial ARCA or ALCA remains controversial, and individualized risk assessment, particularly for those with proximal vessel narrowing or ischemic symptoms, is recommended [21].

In cases with cardiac involvement, advanced imaging modalities may offer additional diagnostic and prognostic value. Cardiac magnetic resonance imaging (MRI) can be utilized to assess myocardial and pericardial involvement, as well as delineate fibroinflammatory tissue with late enhancement [13,22]. Dual-energy CT is another alternative for non-MRI candidates that uses iodine perfusion mapping and spectral curve analysis to analyze myocardial ischemia and fibrosis [23,24]. Furthermore, positron emission tomography-CT is highly sensitive for detecting metabolically active lesions and can guide biopsies and monitor treatment response [25]. Unfortunately, due to the patient’s death shortly after diagnosis, these modalities were not utilized.

Several factors during the clinical course may have contributed to delayed recognition. Due to the initial suspicion for cholangiocarcinoma, the patient underwent a Whipple procedure. In retrospect, one imaging feature that may have favored IgG4-sclerosing cholangitis (IgG4-SC) over cholangiocarcinoma is the presence of long-segment, symmetric narrowing of the distal CBD. This contrasts with the irregular, beaded strictures of primary sclerosing cholangitis or the abrupt, eccentric narrowing of cholangiocarcinoma [26,27]. Distinguishing acute interstitial pancreatitis from AIP or IgG4-pancreatitis can also be challenging due to overlapping imaging features. While IgG4-pancreatitis may present with characteristic features such as “sausage-shaped” enlargement with capsule-like rim and homogeneous delayed enhancement [28], these findings may not be readily apparent in the early stages.

In this case, the pancreatic and biliary findings were initially thought to represent separate processes, but when considered together with the markedly elevated serum IgG4 level, the ACR/EULAR threshold for classification would have been met. By the time a definitive diagnosis was made, the patient had developed progressive multiorgan involvement, with imaging findings alone sufficient to meet classification criteria. Earlier multidisciplinary coordination, expedited retrieval of outside pathology results, and closer monitoring with follow-up imaging may have supported timely immunosuppressive therapy and potentially altered the prognosis.

Taken together, this case exemplifies the complexity of diagnosing and managing IgG4-RD, particularly in its fewer common manifestations. Delayed or missed diagnoses are not uncommon given the wide clinical spectrum. Multidisciplinary collaboration, among various specialties such as gastroenterology, radiology, rheumatology, cardiology, and surgery, is often essential. Ultimately, a high index of suspicion, thorough imaging assessment, and histopathologic confirmation remain the cornerstones of diagnosis. Early initiation of immunosuppressive therapy can favorably impact disease trajectory, but careful monitoring for organ-threatening complications remains essential, especially in cases involving critical organs such as the coronary arteries.

## Patient consent

Written informed consent was obtained from the patient/patient representative for publication of this case report and any accompanying images.
